# Demons-Meigs syndrome: Rare cause of intraperitoneal and pleural effusion

**DOI:** 10.1016/j.radcr.2025.03.033

**Published:** 2025-04-05

**Authors:** Imad Akasbi, Amal Akammar, Zineb Ezzoulali, Hajar Ouazzani chahdi, Ismail Chaouche, Elghali Iraqui houssaini, Meryem Zaryouhi, Nizar El Bouardi, Yassine Belhaj, Layla Tahiri Ousrouti, Badr Alami, MY Youssef Alaoui Lamrani, MY Abdelilah Melhouf, Mustapha Maaroufi, Meryem Boubbou

**Affiliations:** a Radiology Department, Mother and Child Hospital, CHU Hassan II, Sidi Mohammed Ben Abdellah University, Fèz, Morocco; b Adult Radiology Department, CHU Hassan II, Sidi Mohammed Ben Abdellah University, Fez, Morocco; c Gynecology-Obstetrics II Department, CHU Hassan II, Sidi Mohammed Ben Abdellah University, Fez, Morocco; d Department of Anatomic Pathology, CHU Hassan II, Sidi Mohammed Ben Abdellah University, Fez, Morocco; e Laboratory of Biomedical and Translational Research, Sidi Mohamed Ben Abdellah University, Fez, Morocco

**Keywords:** Demons-Meigs syndrome, Ovarian tumor, Hydrothorax, Ascites, CA-125

## Abstract

Demons-Meigs (DM) syndrome is characterized by the association of a benign ovarian tumor most often a fibroma or fibrothecoma with pleural and intraperitoneal effusions. It is a rare pathological entity. We report the case of a 40-year-old female patient with typical DM syndrome, characterized by the coexistence of pleural and intraperitoneal effusions, an ovarian mass, and a CA-125 level of 633 IU/ml. Laparotomy revealed abundant ascites and a large right ovarian mass, which was subsequently removed. Histopathological examination confirmed an ovarian fibroma. DM syndrome has a favorable prognosis, and treatment is primarily based on the removal of the ovarian tumor, without the need for chemotherapy or other therapeutic approaches.

## Introduction

DM syndrome is a condition involving a benign pelvic tumor, typically an ovarian fibroma, associated with pleural and/or peritoneal effusions [[1], [2], [3], [4]]. It remains an exceptionally rare condition, and its pathophysiology is poorly understood [2,5,6]. Imaging techniques such as ultrasound, CT, and MRI play a crucial role in diagnosing DM syndrome by identifying the benign ovarian mass and associated effusions [1,5]. Fibromas and fibrothecomas are the most commonly reported tumors associated with this syndrome. However, before histological examination of the tumor, this rare syndrome often raises concerns about malignancy due to the tumor's size, the presence of ascites, and significantly elevated CA-125 levels [2,6,7].

## Case presentation

**Fig. 1:**
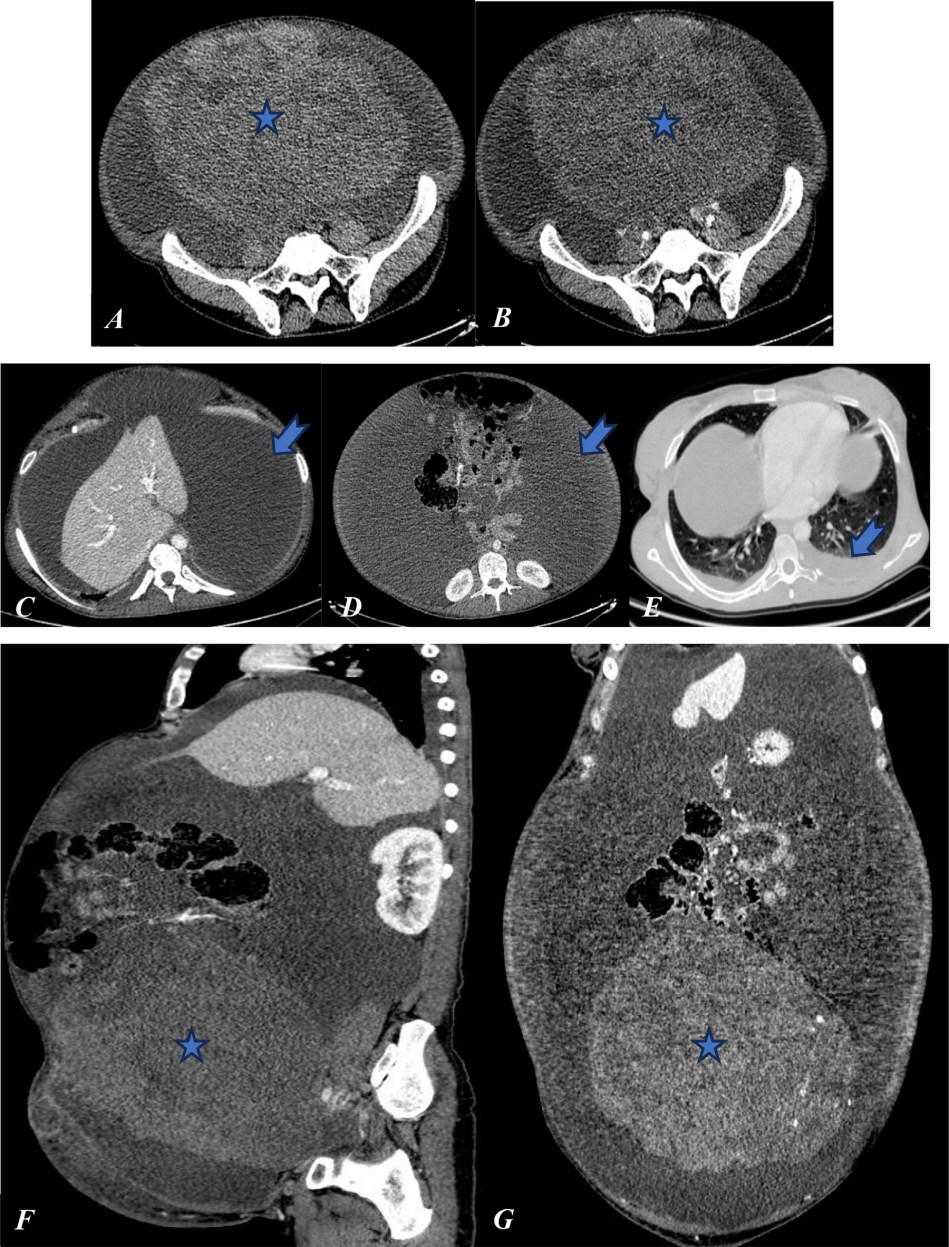
Axial (A, B), sagittal (F), and coronal (G) CT scans showing a large right ovarian mass (blue star). Axial CT scans showing intraperitoneal effusion (C, D) and pleural effusion (E) (blue arrow).

We report the case of a 40-year-old female patient with no significant medical history who presented with abdominal distension, pelvic heaviness, right-sided chest pain, and dyspnea. Clinical examination revealed bilateral pleural effusion, abdominal distension, and a positive fluid wave test. Ultrasound identified a solid-cystic pelvic mass measuring 26 cm in diameter, accompanied by abundant ascites. A thoracic-abdominopelvic CT scan showed a large right ovarian mass with lobulated contours, approximately 28 cm in diameter. The mass exhibited minimal enhancement after contrast administration, consistent with its fibrous nature, and contained calcifications and hypodense areas. No invasion of adjacent structures was observed, and the scan confirmed abundant bilateral pleural and intraperitoneal effusions with no distant metastases (Fig. 1).

**Fig. 2:**
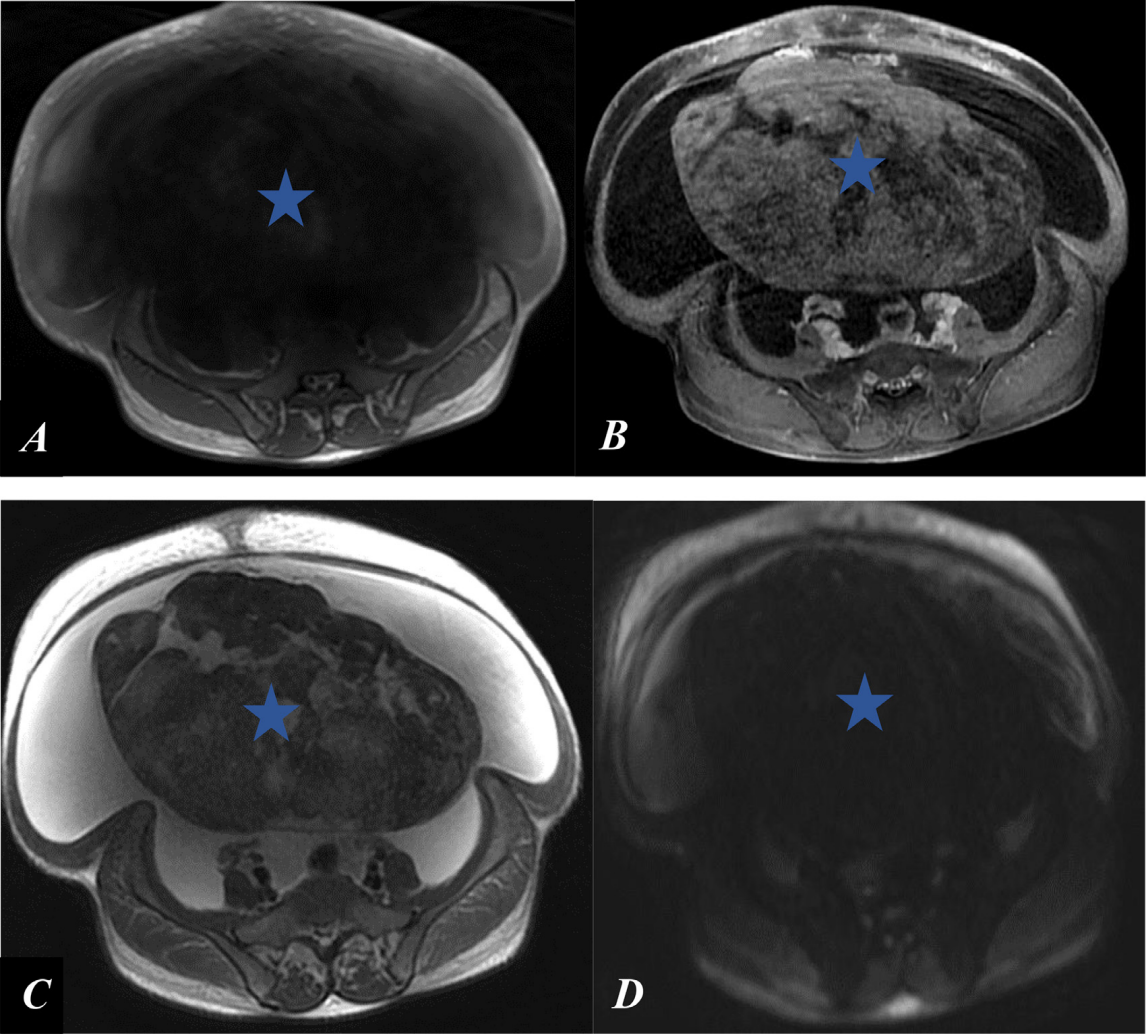
T1-weighted MRI sequence without contrast (A), contrast-enhanced T1-weighted sequence (B), diffusion-weighted sequence (D), and T2-weighted sequence (C) demonstrating a right ovarian mass, hypointense on T1 and T2, nonrestrictive on diffusion, and moderately enhanced after gadolinium administration (blue star).

**Fig. 3:**
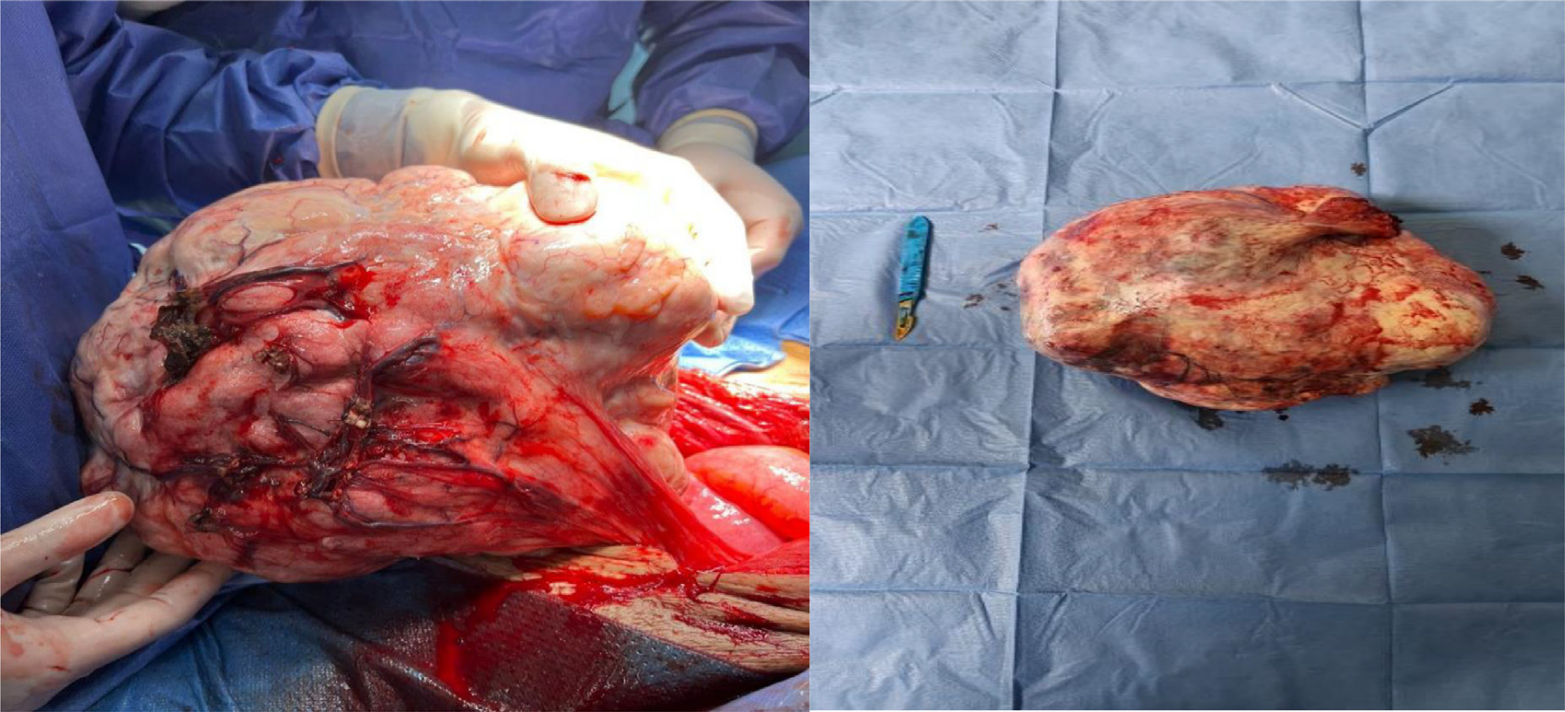
Intraoperative image showing a large right ovarian mass.

**Fig. 4:**
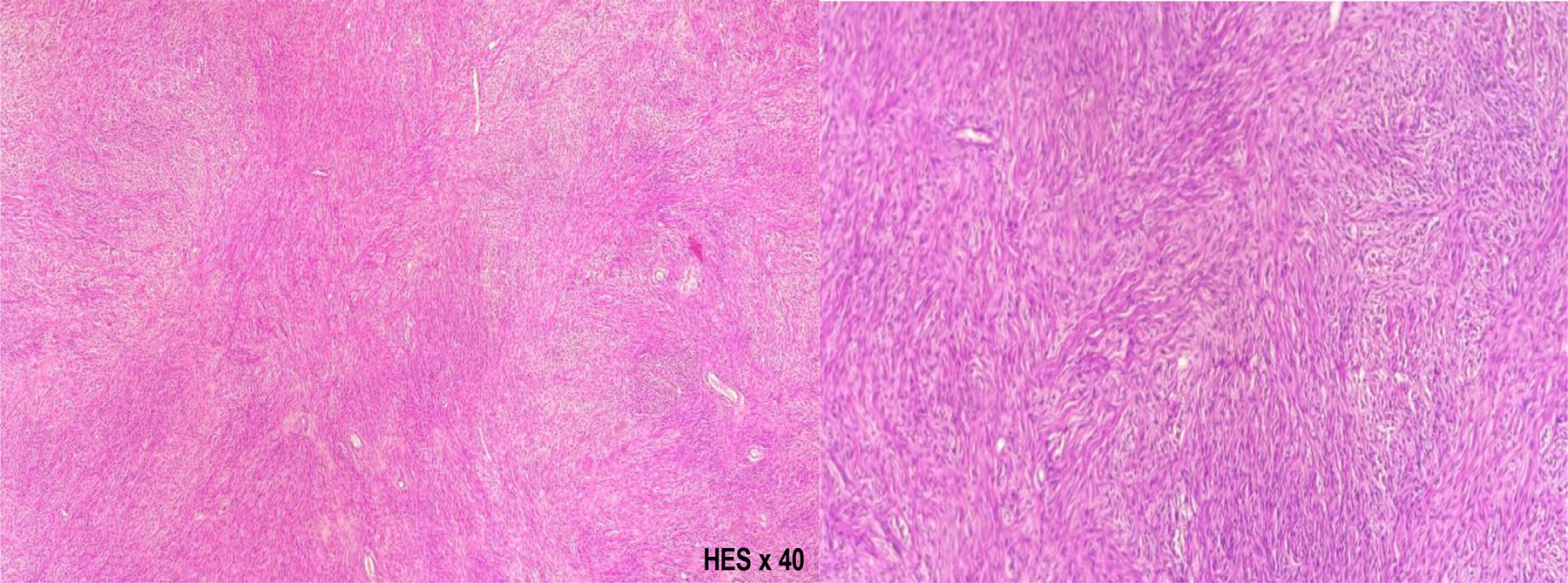
Histological and immunohistochemical findings consistent with an ovarian fibroma.

MRI revealed a large, well-circumscribed, oval-shaped right latero-uterine mass adjacent to the ovary, hypointense on T2-weighted imaging, nonrestrictive on diffusion, and weakly enhanced after contrast administration (Fig. 2). The patient underwent laparotomy, which revealed a large right ovarian mass (Fig. 3) and a significant volume of transudative ascites. The tumor was removed via oophorectomy and omentectomy, and 17 liters of ascites were evacuated. Histological and immunohistochemical findings confirmed an ovarian fibroma (Fig. 4). The postoperative course was uneventful, with complete resolution of pleural and peritoneal effusions observed immediately after surgery, without the need for chemotherapy. CA-125 levels were significantly reduced 6 months after the procedure.

## Discussion

DM syndrome is characterized by the coexistence of a benign ovarian tumor and intraperitoneal and pleural effusions, which typically resolve after tumor removal [[1], [2], [3], [4], [5]]. In 1884, French physician Albert Demons first documented the association of an ovarian tumor with pleurisy and ascites. In 1902, this association was further observed with solid ovarian tumors, uterine fibroids, and broad ligament myomas [2,5,6].

The pathophysiological mechanisms remain poorly understood and largely hypothetical. One mechanical hypothesis suggests that a persistent pleuroperitoneal duct and transdiaphragmatic lymphatic channels play a significant role. According to this theory, tumor pressure on lymphatic vessels causes ascites, which then passes through the duct, leading to pleural effusion [2]. Another hypothesis, the hormonal theory, attributes the condition to endocrine dysfunction from an estrogen-secreting tumor in the genital tract [1,2].

DM syndrome presents with variable symptoms, ranging from incidental discovery during a gynecological examination to pronounced manifestations such as abdominal pain, abdominal distension, and dyspnea associated with pleural effusion [4,6,[8], [9], [10]]. Symptoms vary in intensity, and some cases are considered atypical [3]. The ovarian mass is typically asymptomatic, large, solid, mobile, and unilateral [10].

The hallmark of this syndrome is the complete resolution of ascites and hydrothorax following surgical tumor removal, accompanied by a favorable prognosis [10]. Imaging is critical for diagnosing DM syndrome. Pelvic ultrasound reveals a unilateral, smooth-surfaced, mobile latero-uterine mass. MRI shows a solid, unilateral ovarian mass, typically ovoid or rounded, hypointense on T1- and T2-weighted imaging due to its collagen-rich fibrous composition, nonrestrictive on diffusion sequences, and moderately enhanced after gadolinium administration. CT confirms the diagnosis by characterizing the ovarian mass and detecting peritoneal effusion and hydrothorax, which are often unilateral and predominantly right-sided. These imaging modalities are essential for distinguishing benign lesions from malignant tumors [1,10]. Management of DM syndrome involves tumor excision after confirming its benign nature through imaging and tumor markers [10]. Treatment strategies vary based on the patient's age and fertility goals. In younger women, conservative surgery, such as unilateral oophorectomy or tumor resection, is preferred, while postmenopausal women typically undergo total adnexectomy [10]. The prognosis is excellent, with complete resolution of effusions postsurgery and a life expectancy comparable to that of the general population [10]. Elevated CA-125 levels are not exclusive to malignant ovarian tumors and may be associated with ascites, often correlating with tumor size [1,4,7,[8], [9]].

## Conclusion

Although rare, DM syndrome should be considered by surgeons, as it may mimic advanced ovarian neoplasia. The presence of ascites and/or pleural effusion does not always indicate malignancy in the context of an ovarian tumor. Identifying this syndrome preoperatively allows for more precise surgical planning. Avoiding extensive surgery is critical for this benign condition with a favorable prognosis [7]. Tumor resection effectively resolves ascites and pleural effusion. Imaging plays a vital role in confirming the diagnosis, characterizing the ovarian mass as benign, and visualizing intraperitoneal and pleural effusions [1,10].

## Patient consent

Written informed consent was obtained from the patient for the publication of this case report and any accompanying images. The patient has been assured that all personal information will remain confidential and that identifying details will not be disclosed.

The patient was also informed that this publication is for scientific purposes and has the right to withdraw consent at any time without any impact on their medical care.
